# Engineering a Point-of-Care Paper-Microfluidic Electrochemical Device Applied to the Multiplexed Quantitative Detection of Biomarkers in Sputum

**DOI:** 10.1021/acssensors.3c00523

**Published:** 2023-07-19

**Authors:** Manuel Gutiérrez-Capitán, Ana Sanchís, Estela O. Carvalho, Antonio Baldi, Lluïsa Vilaplana, Vanessa F. Cardoso, Álvaro Calleja, Mingxing Wei, Roberto de la Rica, Javier Hoyo, Arnau Bassegoda, Tzanko Tzanov, María-Pilar Marco, Senentxu Lanceros-Méndez, César Fernández-Sánchez

**Affiliations:** † Instituto de Microelectrónica de Barcelona, IMB-CNM (CSIC), Campus UAB, 08193 Bellaterra, Spain; ‡ Nanobiotechnology for Diagnostics (Nb4D), Institute for Advanced Chemistry of Catalonia (IQAC), CSIC, 08034 Barcelona, Spain; § Centro de Investigación Biomédica en Red de Bioingeniería, Biomateriales y Nanomedicina (CIBER-BBN), 28029 Madrid, Spain; ∥ Centre of Physics of the Universities of Minho and Porto (CF-UM-UP) and LaPMET, 4710-057 Braga, Portugal; ⊥ CMEMS-UMinho, 4800-058 Guimarães, Portugal; # Cellvax, SAS, 94800 Villejuif, France; ∇ Multidisciplinary Sepsis Group, Health Research Institute of the Balearic Islands (IdISBa), 07120 Palma de Mallorca, Spain; ○ Centro de Investigación Biomédica en Red de Enfermedades Infecciosas (CIBER-INFEC), 28029 Madrid, Spain; ◆ Grup de Biotecnologia Molecular i Industrial, Departament d’Enginyeria Química, Universitat Politècnica de Catalunya, 08222 Terrassa, Spain; ¶ Basque Centre for Materials and Applications (BCMaterials), UPV/EHU, 48940 Leioa, Spain; †† IKERBASQUE, 48009 Bilbao, Spain

**Keywords:** point-of-care rapid tests, chronic obstructive pulmonary disease, electrochemical biosensing, paper microfluidics, magnetic nanoparticle-based immunoassay, multiplexed detection

## Abstract

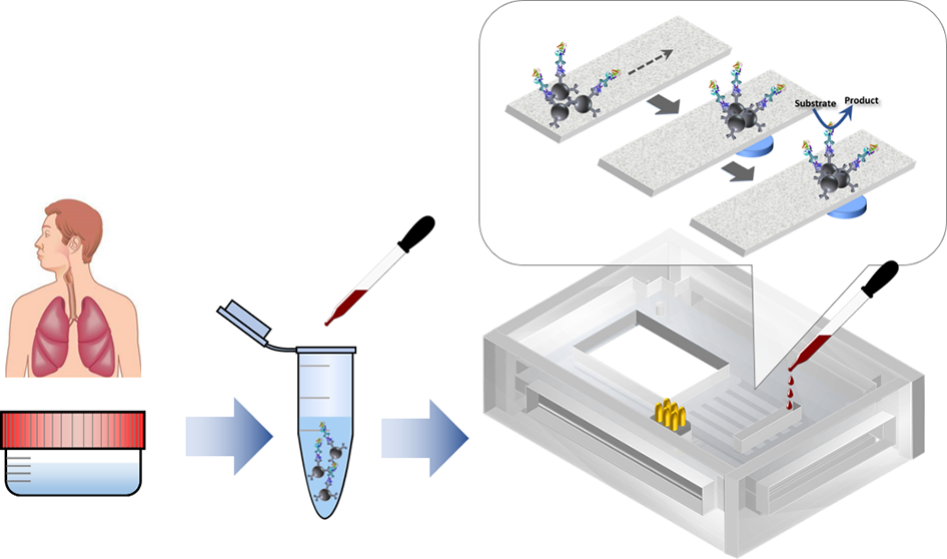

Health initiatives worldwide demand affordable point-of-care devices to aid in the reduction of morbidity and mortality rates of high-incidence infectious and noncommunicable diseases. However, the production of robust and reliable easy-to-use diagnostic platforms showing the ability to quantitatively measure several biomarkers in physiological fluids and that could in turn be decentralized to reach any relevant environment remains a challenge. Here, we show the particular combination of paper-microfluidic technology, electrochemical transduction, and magnetic nanoparticle-based immunoassay approaches to produce a unique, compact, and easily deployable multiplex device to simultaneously measure interleukin-8, tumor necrosis factor-α, and myeloperoxidase biomarkers in sputum, developed with the aim of facilitating the timely detection of acute exacerbations of chronic obstructive pulmonary disease. The device incorporates an on-chip electrochemical cell array and a multichannel paper component, engineered to be easily aligned into a polymeric cartridge and exchanged if necessary. Calibration curves at clinically relevant biomarker concentration ranges are produced in buffer and artificial sputum. The analysis of sputum samples of healthy individuals and acutely exacerbated patients produces statistically significant biomarker concentration differences between the two studied groups. The device can be mass-produced at a low cost, being an easily adaptable platform for measuring other disease-related target biomarkers.

Chronic obstructive pulmonary disease (COPD) is the third leading cause of death worldwide, causing 3.23 million deaths in 2019.^1^ COPD is characterized by an accelerated decline of lung function. Between 20 and 30% of the patients suffer from repeated acute exacerbations (AECOPDs) that present with excessive sputum secretion.

COPD diagnosis is currently confirmed by spirometry standard respiratory function test, which assesses the degree of airway obstruction.^2^ In low- and middle-income countries, where nearly 90% of COPD deaths occur in patients under 70 years of age,^1^ spirometry is often not available, and so the disease is rather poorly diagnosed. The situation worsens when AECOPDs occur because of the lack of sensitive clinical protocols. As a consequence, AECOPD events are diagnosed when they have already occurred. COPD is a highly heterogeneous disease, and AECOPD episodes may also present different etiologies. It has been identified that inflammation is a key underlying mechanism of AECOPDs,^3^ most likely being triggered by bacterial and viral infections. AECOPD entails the secretion by the human immune system of pro-inflammatory cytokines like interleukin-8 (IL-8) and tumor necrosis factor-α (TNF-α), as well as specific enzymes such as myeloperoxidase (MPO).^4,5^ The present study puts the focus on the rapid and simultaneous detection of these three COPD biomarkers, whose sputum concentration levels have previously been correlated with the different stages of the disease.^6−10^ Although enzyme-linked immunosorbent assay (ELISA) is the reference method for the detection of these target biomarkers,^11^ more rapid and reliable multiplexed diagnostic tools are prerequisites in devising earlier and specific strategies for effective COPD therapeutic control. Timely identification of the patient as a frequent exacerbator can be of great help in terms of preparing for emergencies and providing prophylactic relief. Previous studies have revealed that the detection of inflammation biomarkers helps to recognize AECOPDs and even discriminate those due to bacteria from those due to viral agents and noninfectious causes.^12,13^

During the last decades, point-of-care testing (POCT) technology has greatly contributed to the progress in health monitoring.^14^ POCT has become widely available for certain high-incidence diseases, such as the recent COVID-19, thanks to technological advances that have made them more affordable, robust, and user-friendly.^15^ In this context, paper-based analytical devices (μPADs) appear to be very convenient for healthcare diagnostics at the point of need.^16,17^ Like most classical lateral flow tests (LFT) available on the market, paper-microfluidic devices are mostly based on instrument-free qualitative detection of color change or the simple semi-quantitative detection of color intensity using the camera of a mobile device.^18^ However, quantitative readout approaches are highly demanded. One good example is the role that quantitative approaches would have played during the COVID-19 pandemic to aid in efficient patient stratification and timely and effective treatment.^19^ Instrumental electrochemical transduction protocols are more and more widespread in this regard.^20^ Electrochemical transducers and required electronics show well-known advantages for producing quantitative analytical platforms in terms of inherent small size, low cost, low power consumption, portability, high selectivity and sensitivity, as well as the versatility given by the availability of a large number of measuring techniques.^21^ The integration of electrochemical transduction in μPADs has followed two main strategies. On the one hand, electrochemical cells and fluidic components are defined on a single paper substrate. On the other hand, electrochemical cells are manufactured separately on a different substrate and then assembled with the paper fluidic component.^22^ The former inherently aligns both components and puts them in contact using an origami-based folding approach.^23^ In these works, electrochemical cells are mainly produced by screen-printing, but the fabrication and further analytical performance is limited by the porosity and the mechanical robustness of the paper material. The latter stands out for their easier fabrication and much higher flexibility since electrochemical cells can be produced on different substrates, and the paper fluidic component can show any configuration by not being limited by the cell production process.^24^ By contrast, the alignment and pressure required to put in contact the two components should be strictly controlled.

Several μPADs have been developed for the detection of the COPD biomarkers under study in saliva that are based on both qualitative or semi-quantitative detection approaches.^25,26^ There are also examples of electrochemical transduction approaches applied to, for instance, the detection of salivary concentrations of IL-8^27,28^ and TNF-α.^29,30^ Although the use of saliva is advantageous owing to sampling simplicity, the very low concentrations of biomarkers present in this matrix^31^ require the development of highly sensitive devices. Sputum is also considered a noninvasive sample that, unlike blood and saliva, contains higher concentrations of biomarkers providing direct information on the patient’s lung state^32^ and making it highly relevant in the early diagnosis of COPD and AE episodes. A simple point-of-need LFT has been reported for the rapid visual detection of MPO in diluted sputum using anti-human-MPO antibodies printed onto nitrocellulose membranes.^33^ Previous approaches for detecting MPO, IL-8, or TNF-α in sputum were mainly based on ELISA.^9^ However, none of them relies on μPAD architectures that could potentially be used as POCT devices. These three biomarkers studied individually are not representative of COPD/AECOPD onset and progression, while they may be indicative of more than one disease.^34^ For instance, the secretion of IL-8 could also be related with cancer, COVID-19, sepsis, or cystic fibrosis. Therefore, in order to avoid misdiagnosis, multiplexed detection of biomarkers, that is, simultaneous detection of multiple biomarkers in a single assay, is strongly needed to improve diagnostic accuracy.^35^

Here, we describe the particular design and fabrication of a portable, versatile microfluidic electrochemical device that combines three main parts. These are an electrochemical transducer comprising a reusable array of miniaturized two-electrode cells for multiplexed electrochemical detection and a disposable paper fluidic component engineered to be easily handled and aligned with the electrochemical transducer in a polymeric cartridge, which in turn enables the rapid replacement of the paper-microfluidic component after each analysis. We combine it with enzyme-based immunoassay approaches performed on magnetic nanoparticles for sample pretreatment, including biomarker preconcentration, which greatly facilitates working with very complex physiological matrices.^36−38^ The paper component enables the placement and concentration of the nanoparticles in an area close to the electrochemical transducer, together with the fluid handling required for the final detection by chronoamperometry.^39^ We show the simplicity of this platform in terms of fabrication and performance for carrying out the simultaneous quantitative detection of IL-8, TNF-α, and MPO inflammatory biomarkers present at certain concentrations in human sputum samples upon the occurrence of COPD-related AE events.

## Results

### Device Concept and Fabrication

**Figure 1:**
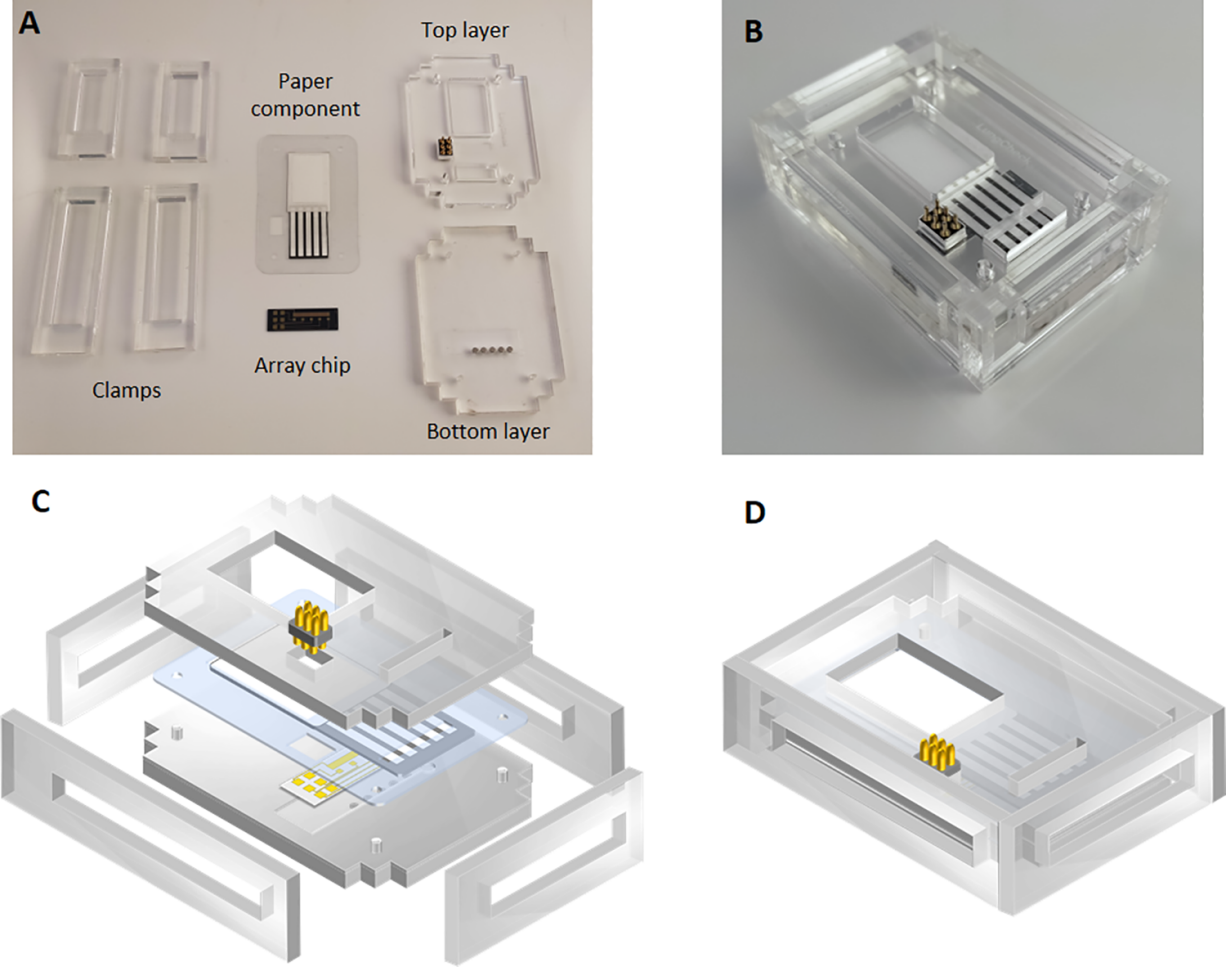
Components and assembly of the paper-microfluidic electrochemical device. (A) Photograph of the individual device components, including the two-electrode array chip containing five electrochemical cells and the paper component with five fluidic channels patterned by wax printing and the different parts of the PMMA cartridge. A spring-loaded connector and five neodymium magnets are inserted in the top and bottom layers, respectively. (B) Photograph of the assembled device, whose dimensions (*W* × *L* × *H*) are 48 × 67 × 19 mm^3^. (C) Scheme of the different components being aligned and assembled to construct the (D) overall device.

Engineering a multiplexed device for timely quantitative detection of several target analytes that could potentially be applied at the point of need required working on simple technological approaches and low-cost materials that were not detrimental to the overall device performance. Our aim was to take advantage of (i) the sensitivity and versatility of electrochemical techniques, (ii) the feasible miniaturization of the electrochemical transducers together with compactness and low-power instrumentation requirements, as well as (iii) the excellent physicochemical properties of high-quality paper-microfluidic substrates in terms of material inertness and liquid flow by capillary action. The latter is one of the most relevant features when working in paper microfluidics since no external actuating components are required to drive the liquid inside the fluidic system, making the overall platform more amenable to be compact and easily deployed. In this context, an approach combining a miniaturized two-electrode cell array and a purposefully defined multichannel paper fluidic component was developed to fit in a cartridge that allowed both parts to be easily aligned against each other and to be replaced if necessary (Figure 1).

The transducer array’s particular design contained five 2-electrode electrochemical cells sharing a common counter/reference electrode (CRE) that minimized the overall area of the silicon chip by reducing the number of required electrical tracks and contact pads (Figure 1A).

The five electrochemical cells could be individually addressed without showing any chemical or electrical cross-talk. We previously showed the excellent performance of the chip in the detection of the activities of different enzymes whose reactions were coupled to reversible redox mediators.^40^ Among them, the activities of horseradish peroxidase (HRP) and MPO were tested. In the present work, HRP was used as the label of the immunoassays implemented in the device for the detection of TNF-α and IL-8, whereas MPO was also selected as a biomarker to be detected in sputum, as explained in detail in the following sections.

The paper component included five fluidic channels patterned by a wax printing process^41^ and a laser-cut sink pad (Figure 1A). As a disposable component, this paper enables the sequential additions of the MNPs dispersion upon sample pretreatment, washing steps, and measuring solution to be performed without the need for any external mechanical components. Among the different paper patterning approaches that have been reported,^42^ wax printing has been widely applied to the simple parallel fabrication of a variety of features.^43^ This technique involves a three-step method that includes pattern drawing, paper printing using a commercial wax printer, and a thermal step carried out at a low temperature using an oven or a hot plate to allow impregnation of the wax across the paper to reach the back-paper side. Several papers of filter and chromatographic quality were tested as well as different fluidic configurations that include channel and sink pad geometries and dimensions. Whatman 1 paper of chromatographic quality, commonly used in paper microfluidics, was eventually selected, looking at the effective flow of the different solutions and the liquid volume capacity required to carry out the analyses. The channels and sink pad of the paper fluidic component were positioned and sandwiched between two sticky vinyl layers. A simple, transparent vinyl material was selected that could be easily patterned by blade cutting. The two layers included several opened windows to access the paper in the sample addition, transducer, and evaporation areas, as well as to provide access to the on-chip contact pads by the spring-loaded connectors (Figure S1, Supporting Information (SI)).

The polymeric cartridge also included several features to easily insert and align the transducer and the paper fluidic components (Figure 1A,C). Several cartridge designs were assessed, paying special attention to the alignment and contact between the transducer array and the paper fluidic component, as well as the device opening and closing. The final design included four rigid clamping structures that pressed the bottom and top parts of the cartridge, making a frame that could be rapidly assembled and disassembled when necessary. Likewise, the dimensions of these structures ensured that both components were in intimate contact and that a constant pressure was applied against each other in the five sensing areas of the device.

Analysis of complex biological samples, such as sputum, with POCTs proved to be difficult. For this, a sample pretreatment protocol that made use of antibody-modified magnetic nanoparticles was implemented in order to capture, label, and preconcentrate the target biomarkers in a proper buffer solution (Figure S2A, SI). The MNP diameter (200 nm) was selected after testing their efficient flow on the selected chromatographic paper and their effective entrapment and concentration with 2 mm diameter Nd magnets located below each paper channel 1.5 mm upstream from the position of the working electrodes (Figure 1A). The complete analytical procedure comprised six steps: (i) sample addition to the solution containing the capture antibody-modified MNPs and the corresponding detection antibody, if necessary, (ii) MNP washing upon biomarker capture, (iii) MNP resuspension in a buffer solution, (iv) MNP addition to the device inlet, (v) paper channel washing and, (vi) addition of enzyme–substrate solution to carry out the chronoamperometric detection (Figure S2B,C, SI). All of them are simple pipetting processes for which no specific training is needed. Some 100 μL of the sample solution was required, which was added to 400 μL of the MNP suspension. The 500 μL volume was reduced to 100 μL upon MNP washing and resuspension. Of this, some 5 μL was added to a single paper channel. Some 5 μL volumes were also used for the washing and enzyme reaction steps.

### Magneto-Immunoassay Studies

Two different immunoassay formats were implemented on the MNPs to carry out the detection of the three target biomarkers (Figure S2A, SI). The detection of MPO was based on the measurement of the enzyme activity upon being captured with anti-MPO-modified MNPs. The detection of IL-8 and TNF-α was based on a sandwich-like immunoassay format using MNPs modified with the capture antibodies to the corresponding biomarkers and detection antibodies conjugated to HRP enzyme. The applied optical and electrochemical detection protocols were based on measuring the MPO and HRP activities in solutions containing H_2_O_2_ substrate and an appropriate enzyme redox mediator.

First, carboxylated MNPs were functionalized by covalent immobilization of the anti-TNF-α, anti-IL-8, and anti-MPO capture antibodies, and conjugation efficiencies of 97.3, 95.6, and 95.6%, assessed by Bradford analysis, were achieved, respectively. The immunoassays were then optimized in 2D assays in round-bottom 96 microplates. Studies to select the amount of the MNPs and concentration of detection antibody–HRP conjugates were carried out using colorless 3,3′,5,5′-tetramethylbenzidine (TMB) as the redox mediator for both HRP and MPO enzymes and measuring the absorbance of the products of the corresponding enzyme reactions at 450 nm (Figure S3, SI). A concentration of 187.5 μg/mL conjugated MNP for the three target analytes in combination with 2 μg/mL detection antibody–HRP conjugate (for IL-8 and TNF-α) produced the highest signals. Then, a study to estimate the analytical performance of the magneto-immunoassays, using the previously optimized conditions, was carried out at different concentration ranges of the three target analytes, repeating the measurements in two consecutive days. The change in the absorbance values with the target analyte concentration was plotted and adjusted to a dose–response semi-logarithmic curve (Figure S4, SI). The results show concentration ranges of 125–2000 pg/mL IL-8, 95–3000 pg/mL TN-α, and 10–2000 ng/mL MPO could be suitable for the calibration of the electrochemical devices, also providing a rough estimate of the sensitivity that should be achieved when these magneto-immunoassays were implemented in the final device.

Possible matrix effects that might arise due to the sputum sample composition were also assessed here using an artificial sputum solution. The first study was carried out with the two cytokines. When the biomarkers were measured in the undiluted solution, the magneto-immunoassay sensitivity decreased by more than 50% with respect to that observed in the standard buffer solution. However, this effect was minimized when the artificial sputum was diluted 1:1 in the working buffer (PBST), retaining about 100% of the analytical signal. A similar study was carried out with MPO but just in the 1:1 diluted artificial sputum showing the same trend as that of the other two biomarkers (Figure S5, SI).

### Analytical Performance of the Electrochemical Device

**Figure 2:**
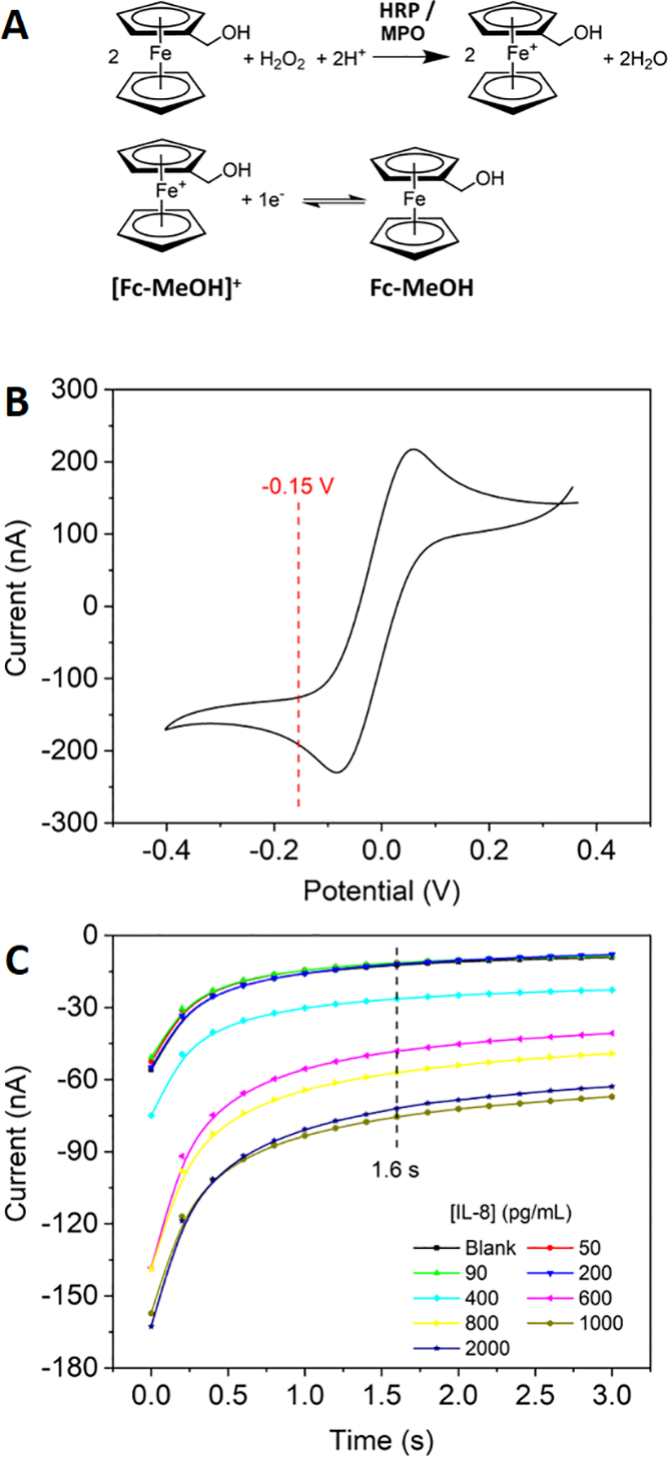
Electrochemical detection of biomarkers. (A) Reactions involved in the detection using ferrocenemethanol (Fc-MeOH) as redox mediator for both MPO biomarker and HRP label. The ferrociniummethanol cation [Fc-MeOH]^+^ generated by the enzymes is reduced back to Fc-MeOH at the electrode surface. (B) Cyclic voltammogram recorded with a single on-chip two-electrode electrochemical cell in an acetate buffer pH 4.5 solution containing both Fc-MeOH and [Fc-MeOH]^+^ at a scan rate of 50 mV/s. A set potential of −0.15 V was chosen for the subsequent chronoamperometric measurements. (C) Representative raw chronoamperometric responses recorded for 0 to 2000 pg/mL IL-8 solutions. The current responses recorded at 1.6 s were used as analytical signals.

A schematic illustration of the overall analytical protocol implemented in the electrochemical device is shown in Figure S2C, SI. Electrochemical measurements were based on the use of ferrocenemethanol (Fc-MeOH) redox mediator for both MPO biomarker and HRP label (Figure 2A). The corresponding enzyme reactions produced ferrociniummethanol cation [Fc-MeOH]^+^ that was easily detected by carrying out chronoamperometric measurements at a set potential at which [Fc-MeOH]^+^ was reduced back to Fc-MeOH, using the two-electrode electrochemical cells defined on the transducer array chip.^40^ First, cyclic voltammetric (CV) experiments were performed on the electrochemical device by adding a certain volume of an acetate buffer solution containing both Fc-MeOH and [Fc-MeOH]^+^ in order to characterize the redox process of this reversible redox pair and to find the best conditions for recording the device analytical signal by chronoamperometry (see the SI for more experimental details).

Figure 2B shows a representative CV curve, showing the anodic and cathodic faradic processes taking place between the two electrodes in the cell. These results were quite similar to those obtained when the redox pair was measured with the same transducer in batch conditions,^40^ indicating that the transducer array electrochemical performance was not affected by the incorporation of the paper fluidic component. The half-wave potential (*E*_1/2_) was close to 0 V, this being the observed behavior when both WE and CRE were made of the same material and were involved in the same reversible electrochemical reactions.^40^ From this CV, a set potential of −0.15 V was chosen to carry out chronoamperometric measurements to detect the [Fc-MeOH]^+^ generated by the HRP and MPO enzyme activities. This overpotential ensured that small shifts in the CRE half-cell potential due to changes in the solution concentrations of both Fc-MeOH/[Fc-MeOH]^+^ species upon the corresponding enzyme reactions would have a negligible influence on the recorded current at the working electrode, as previously demonstrated.^40^

**Figure 3:**
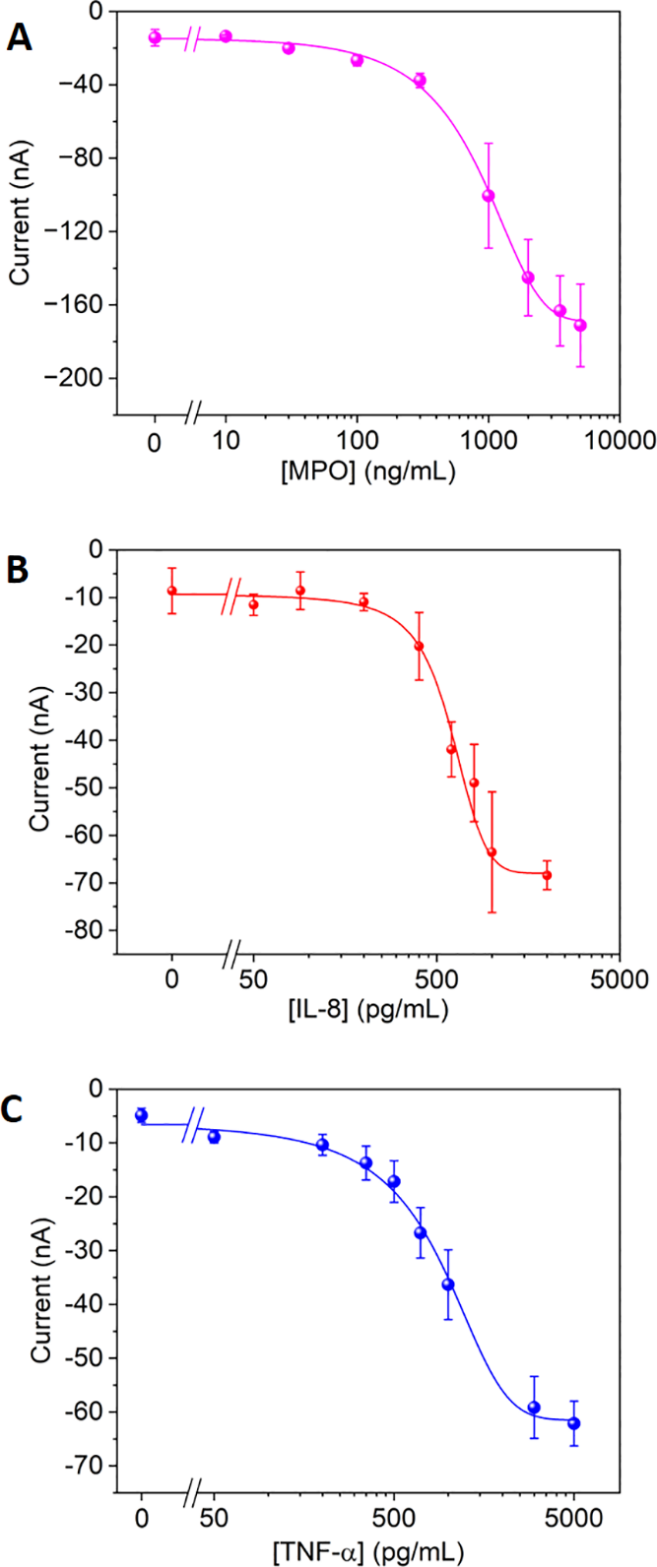
Dose–response calibration curves in the semi-logarithmic plot. The current responses recorded at 1.6 s were used as analytical signals. Each data point represents the mean value of three replicates carried out consecutively for (A) MPO, (B) IL-8, and (C) TNF-α biomarkers. The standard deviation values of these replicates are represented as error bars.

Then, the analytical performance of the paper-microfluidic electrochemical device was initially assessed in standard buffer solutions containing just one biomarker at a time. This study provided the estimated values of the analytical parameters for the three biomarkers. In all cases, the chronoamperometric responses to four different biomarker concentrations plus the blank signal were simultaneously recorded using the five channels included in the device. A total of eight biomarker concentrations plus two blanks were assayed, and for this, two paper fluidic components were used to carry out a complete calibration curve. The raw chronoamperometric response profiles for different biomarker concentrations were recorded (Figures 2C and S6, SI). The corresponding calibration curves were constructed using the current responses recorded at 1.6 s for the three biomarkers (Figure 3). Semi-logarithmic dose–response curves showing the common sigmoidal trend observed when working with immunoassays were plotted. The analytical parameters, extracted from the curve fittings, are summarized in Table 1. The overall analysis time was around 75 min, representing a time reduction of around 45 min in comparison with the optical (absorbance) immunoassay protocol that did not compromise its analytical performance. Moreover, the liquid volumes required for carrying out the electrochemical detection were greatly reduced by more than 90% while keeping the same number of manual steps. The number of steps and the sample volume could be easily reduced by slightly adjusting the implemented immunochemical reactions. Indeed, the simultaneous incubation of the capture antibody-modified MNPs, together with the respective detection antibody conjugate and the sample, would reduce the number of required washing steps. Likewise, the sample volume could be decreased by one-fourth, considering that just 5 μL of the MNP suspension upon capture of the biomarker was added to each paper channel of the device and used for each measurement. This, in turn, translates into a lower cost per analysis, which was estimated to be below 1 €.

**Table 1:** Analytical Parameters Obtained from the Calibration Curves for Each Biomarker^a^

biomarker	concentration range	*R*^2^, *N* = 3^b^	LD^c^	EC_50_^d^
IL-8	0–2000 pg/mL	0.982	420 pg/mL	624 (80) pg/mL
TNF-α	0–5000 pg/mL	0.996	174 pg/mL	1070 (390) pg/mL
MPO	0–5000 ng/mL	0.994	116 ng/mL	1044 (292) ng/mL

a The dose–response semi-logarithmic curves from Figure 4 were fitted using the GraphPad Prism software. The standard deviation values of the three replicates are indicated in brackets.

b N: number of replicated calibrations.

c LD: limit of detection calculated using the 3σ IUPAC criterion.

d EC_50_: half-maximal effective concentration.

A study of the potential matrix effect on the analytical signal was carried out using artificial sputum diluted 1:1 in PBST following the previous results recorded with the magneto-immunoassay based on absorbance detection (Figure S5, SI). Results were quite similar, and a decrease in the slopes of the linear range of 15% for MPO, 12% for IL-8, and 10% for TNF-α was observed (Figure S7, SI). At this point, it should be noted that real sputum samples were not analyzed directly because a biomarker extraction process was required. Therefore, the effect of the matrix composition on the device’s analytical sensitivity was expected to be minimized and likely to be negligible.

### Multiplexed Performance of the Electrochemical Device

The multiplexing capabilities of the device were assessed by measuring the three biomarkers simultaneously with the device. This required individually incubating three aliquots of the sample with MNPs modified with the capture antibodies to a single target biomarker, which could then be sequentially added to individual paper channels of the device and eventually analyzed simultaneously by measuring the HRP label and MPO activities using the enzyme solution containing the Fc-MeOH redox mediator.

For this, a standard solution containing the three biomarkers at concentrations close to the estimated EC_50_ values was prepared (Table 1). Simultaneous measurements of the three biomarkers plus two blanks were carried out following the same protocol as above. The recorded current values were used to estimate the biomarker concentrations by interpolation in the respective calibration curves. As can be observed in Table 2, the estimated biomarker concentrations lie within the real concentration values considering the standard deviation (*N* = 3). The accuracy of the paper-microfluidic electrochemical device using the estimated mean value of the three replicates was evaluated in terms of the recovery percentage, this being above 83% for the three biomarkers.

**Table 2:** Multiplexed Performance of the Electrochemical Device^a^

biomarker	added concentration	interpolated concentration	recovery (%)
IL-8	600 pg/mL	583 (67) pg/mL	97.2
TNF-α	1000 pg/mL	893 (199) pg/mL	89.3
MPO	1000 ng/mL	831 (271) ng/mL	83.1

a The recorded current signals at 1.6 s were used to interpolate the concentration values in the respective calibration curves. The standard deviation values of three replicates are indicated in brackets.

### Analysis of Human Sputum Samples

Eight human sputum samples, three collected from healthy individuals and five from COPD patients, were pretreated, as explained in the Materials and Methods section (SI). Samples were highly viscous and required a biomarker extraction process before analysis.^44^ The extracts were analyzed using the electrochemical device for the detection of three biomarkers separately. First, the chronoamperometric responses to the three samples from healthy individuals plus the blank signal were simultaneously recorded using the five channels included in the device. Then, the five extracts from COPD patients were analyzed in the same way.

The difference in the device analytical signal for the three biomarkers between both sets of samples was clearly significant (Figure 4). A high probability (Student’s *t*-test **P < 0.01 for MPO and TNF-α, ***P < 0.001 for IL-8) was obtained to distinguish healthy individuals from those suffering from the disease. Current intensities recorded in samples from healthy individuals do not differ from those of the blanks. By interpolating in the respective calibration curves and considering the weight of sputum used to obtain each extract (Table S1, SI), the concentration of each biomarker per sample was estimated (Table S2, SI). It should be noted that the current signals of the sample extracts from healthy individuals were lower than the values recorded in the calibration studies, meaning that the biomarker concentrations may be below the limits of detection of the device.

**Figure 4:**
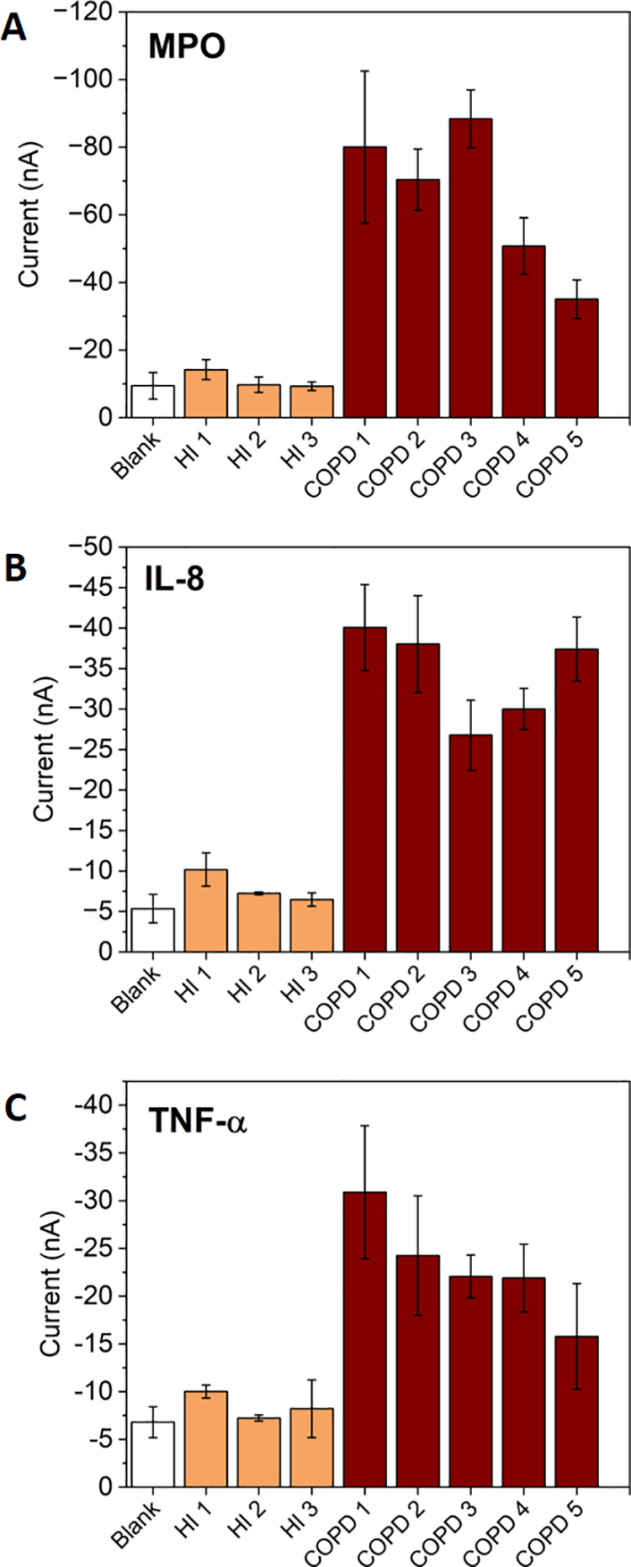
Bar graphs showing the analytical current signals obtained in the analysis of the human sputum samples with the electrochemical device. “HI” corresponds to extracts from healthy individuals, and “COPD” corresponds to extracts from COPD patients. The analytical signals correspond to the mean current values of the chronoamperometric signals recorded at 1.6 s for (A) MPO, (B) TNF-α, and (C) IL-8. The standard deviation values of three replicates carried out consecutively are represented as error bars.

These results show that the device unambiguously detected the three biomarkers in real sputum samples and was able to discriminate between healthy individuals and those suffering from the disease. It also points out that the sample pretreatment procedures carried out to extract MPO enzyme on the one hand and IL-8 and TNF-α on the other hand were highly suitable for our purposes.

## Discussion

Inflammation plays an important role in the pathogenesis of COPD and has been recognized as a key underlying mechanism of AECOPD, mainly triggered by bacterial and viral respiratory infections. The selection in this work of two pro-inflammatory cytokines like IL-8 and TNF-α, and MPO enzyme is based on previous studies on the clinical significance of these target biomarkers for rapidly detecting AECOPD and further discriminating events caused by bacteria, viral agents, and noninfectious causes. Activated neutrophils excreted IL-8, and it has been shown that its sputum concentration increases during AE events.^10^ TNF-α is expressed by activated macrophages and neutrophils, and it has been reported that its concentration in sputum increases up to fourfold during AE compared with that of healthy individuals.^45^ MPO is also released by neutrophils, producing hypochlorous acid bactericide.^46^ The device presented here not only detects the presence of MPO in sputum samples but also electrochemically measures the MPO activity using an appropriate redox mediator.

Clinically relevant concentration ranges of the selected target biomarkers are yet to be established.^9^ According to the literature, sputum IL-8 concentration can vary from 228 pg/mL in healthy individuals to more than 7 μg/mL in bacterial AE of COPD patients.^47^ In the case of TNF-α, the sputum concentration can vary from zero in healthy individuals to 135 pg/mL in COPD patients,^48^ while the sputum MPO concentration can be within 0.2 μg/mL in healthy individuals to 9.1 μg/mL in COPD patients.^49^ The combined quantitative detection of these three biomarkers could shed more light on their clinical significance for reliable AECOPD diagnostics combined or correlated with other clinical variables such as patient symptoms, disease severity, and results of pulmonary function tests carried out by the analysis of breath with airflow meters and portable spirometers.^10^ It could also aid in the prediction of clinical outcomes such as time of hospitalization, response to treatment, recovery, AE frequency, and etiology.

The deployed implementation of compact analytical platforms that enabled the quantitative simultaneous detection of several biomarkers could have a huge impact on effective disease management by improving the underdiagnosis and misdiagnosis rates and in turn alleviating the economic burden on the healthcare systems. The situation is even more dramatic in low and middle-income countries, where 80% of COPD cases get undiagnosed in clinical care.^50^

WHO Noncommunicable Disease Global Action Plan addressed the necessity for diagnostic tools for noncommunicable diseases that were affordable, reliable, and safe and showed improved disease diagnostic capacity and human resources.^51^ In 2003, WHO coined the well-known ASSURED criteria (affordable, sensitive, specific, user-friendly, rapid and robust, equipment-free, and ready to be delivered) that a diagnostic tool in the developing world should fulfill to guide disease management effectively. This is also an excellent criterion for a wide number of rapid diagnostic tests that could be performed at the point of need. New analytical approaches should try to address these needs and be benchmarked against them.

Bearing in mind all of these aspects, we put the focus on developing a compact analytical tool integrating a miniaturized array of electrochemical transducers and a paper fluidic component combined with immunoassays to the three target biomarkers performed on magnetic nanoparticles. The device was engineered so that it can be produced at a low cost (affordable). It enables the quantitative simultaneous measurement of the three target biomarkers in 75 min (rapid) at the concentration levels that appeared to be clinically relevant, as shown by the analysis of sputum samples (sensitive and specific). Up to six steps are required for the performance of the device, mostly based on volumetric controlled addition of different solutions, which requires minimum technical training (user-friendly).

We looked at making the device sustainable and keeping the cost per analysis low in a way that the electrochemical transducer could be reused for a large number of measurements while the paper component was easily replaced following every single measurement. This did not compromise the device performance, nor did it pose any risk to the user in terms of sample and solution contamination. The sample pretreatment process was carried out outside the platform in tubes and the paper component, onto which several solutions flow and could easily be manipulated, as it was laminated into two vinyl layers that just exposed those areas required for solution addition, electrochemical measurement, and solution evaporation. Moreover, the paper component could be disposed of by incineration.^52^ The electrochemical transducer could be mass-produced using microelectronic technologies on silicon wafers, while the mass fabrication of the disposable paper component could be achieved by easily scalable rapid prototyping techniques. Therefore, all of the components are produced with well-established technologies, so a high fabrication yield and a low device-to-device variability can be achieved (robust). A cost for the production of small quantities of the transducer and the paper component of 20 € and 5 € per unit was estimated.

The device can be controlled by a battery-powered instrumentation that could be connected to a mobile device (not equipment-free). While commercial electronics were used for all of the analytical studies included in this work, compact tailor-made instrumentation has already been fabricated for evaluating the simultaneous performance of up to eight electrochemical cells. It is a portable 85 × 78 × 45-mm^3^ amperometer device (Figure S8, SI) that shows a measurement range of 0–4 μA and a resolution of 2 nA; it is powered by a battery and easily controlled by a software designed in LabVIEW. Variabilities of less than 5% in the responses of the electrochemical transducer in standard solutions of a representative redox species were recorded compared to those of the commercial electronics. These electronics, combined with the presented electrochemical paper device, could result in the production of a self-contained analytical tool that could potentially be deployed and used at the point of need.

While the use of immunoassays performed on MNPs may appear to reduce the applicability of the device, these are very useful when working with complex samples that have to be treated to extract the target biomarkers. An initial study on the stability of the antibody-modified MNPs evidenced that they were functional for at least three months when stored in solution in the refrigerator at 4 °C. The HRP and MPO substrate solution containing H_2_O_2_ and Fc-MeOH redox mediator was prepared on a daily basis, and its stability was not further studied. However, commercial solutions similar to this one that can be stored at 4 °C for months are available and used routinely in ELISAs.

The minimally invasive collection of sputum samples was considered. Sputum’s particular physical properties and complex chemical composition require target biomarker extraction using protocols routinely applied in clinical laboratories. These include the addition of chemical reagents and solutions as well as centrifugation steps that could not be avoided. For this, a minimum infrastructure should be available when working with this biological medium. It has recently been reported that the collected sputum samples could also be stored and dried at ambient conditions for up to 10 days using a technology developed by A. Dsouza et al., thus facilitating sputum transportation if necessary.^53^ Nevertheless, sample processing has to be carried out upon sputum reconstitution and before carrying out the analyses, too. The device quantitatively detected the three target biomarkers at those concentrations that appeared to be clinically relevant. This was confirmed by measuring the biomarkers in eight sputum samples of healthy individuals and those suffering from AECOPD, obtaining statistically significant differences between the two sample groups (Student’s *t*-test **P < 0.01 for MPO and TNF-α, ***P < 0.001 for IL-8).

Technical approaches for alleviating sample manipulation and decreasing the number of steps while keeping the correct analytical performance of the device could be tackled. MNPs modified with the capture antibodies to the different target biomarkers could be mixed and incubated with just one sample aliquot. Likewise, detection antibodies labeled with HRP for each biomarker could be incorporated on individual channels of the paper component, following a similar strategy to that of routinely used lateral flow immunoassay approaches. Upon sample incubation, MNPs could be added to the paper component, allowing them to flow and react with the detection antibody conjugates on each paper channel for the individual detection of the target biomarkers in the device. The further incorporation of all of the required reagents in the paper component while working on device storage stability and achieving an ideal sample-in answer-out performance is being addressed. Lyophilization reagent procedures could aid in the incorporation of the different biocomponents, while the integration of some simple actuating structures on the paper component may allow for the sequential performance of the required assay steps in a semi-automatic fashion.^42^ This might bring the presented technology closer to the so-called target product profiles defined for different analytical platforms applied in the diagnostic of diseases under specific conditions of use.^54^

## Conclusions

Overall, the results of this work highlight the potential of the presented analytical platform based on the particular combination of paper microfluidics, electrochemical detection, and enzyme-based immunoassays performed on magnetic nanoparticles. Having five independent measuring channels, the device can simultaneously measure the three biomarkers in a sputum sample plus one blank and one control solution, with the user just having to perform several manual steps. The device performance and its simple architecture point out that it could be applied as a point-of-care device, aiming at improving AECOPD diagnostics and advancing toward the so-called personalized medicine.

## Supporting Information Available

Materials and Methods section, optimization of the magneto-immunoassays with optical detection and analytical performance, study of matrix effects and raw chronoamperometric device responses, estimation of the concentration of each biomarker per sputum sample, description of compact tailor-made instrumentation, and comparison with commercial electronics (PDF)

## Notes

M.G.-C., An.B., and C.F.-S. are listed as inventors on a patent describing this technology: PCT/EP2021/071324 (Biosensor system for multiplexed detection of biomarkers).

## Abbreviations

AECOPD/AE: acute exacerbations of COPD
COPD: chronic obstructive pulmonary disease
CRE: counter/reference electrode
CV: cyclic voltammetry
EC_50_: half-maximal effective concentration
ELISA: enzyme-linked immunosorbent assay
Fc-MeOH: ferrocenemethanol
[Fc-MeOH]^+^: ferrociniummethanol cation
HRP: horseradish peroxidase
IL-8: interleukin-8
LD: limit of detection
LFT: lateral flow test
MNPs: magnetic nanoparticles
μPADs: paper-based microanalytical devices
MPO: myeloperoxidase
NCDs: noncommunicable diseases
PBS: phosphate-buffered saline solution
PBST: PBS with 0.05% Tween 20
POCT: point-of-care testing
TMB: 3,3′,5,5′-tetramethylbenzidine
TNF-α: tumor necrosis factor-α
WE: working electrode
WHO: world health organization
