# Supplementary material for: Engineering a Point-of-Care Paper-Microfluidic Electrochemical Device Applied to the Multiplexed Quantitative Detection of Biomarkers in Sputum
Source: ACS Sens. 2023 Jul 19;8(8):3032–42. doi: 10.1021/acssensors.3c00523 (PMC10463273; doi:10.1021/acssensors.3c00523)
Supplement: Supplementary file 1 — se3c00523_si_001.pdf [file se3c00523_si_001.pdf]

Supporting Information

Engineering a Point-of-Care Paper-Microfluidic Electrochemical Device Applied to the Multiplexed Quantitative Detection of Biomarkers in Sputum

Manuel Gutiérrez-Capitán[1], Ana Sanchís[2,3], Estela O. Carvalho[4], Antonio Baldi[1], Lluïsa Vilaplana[2,3], Vanessa F. Cardoso[4,5], Álvaro Calleja[1], Mingxing Wei[6], Roberto de la Rica[7,8], Javier Hoyo[9], Arnau Bassegoda[9], Tzanko Tzanov[9], María-Pilar Marco[2,3], Senentxu Lanceros-Méndez[4,10,11], and César Fernández-Sánchez[1,3]*

1 Instituto de Microelectrónica de Barcelona (IMB-CNM), CSIC, 08193 Bellaterra, Spain
2 Nanobiotechnology for Diagnostics (Nb4D), Institute for Advanced Chemistry of Catalonia (IQAC), CSIC, 08034 Barcelona, Spain
3 Centro de Investigación Biomédica en Red de Bioingeniería, Biomateriales y Nanomedicina (CIBER-BBN), 28029 Madrid, Spain
4 Centre of Physics of the Universities of Minho and Porto (CF-UM-UP) and LaPMET, 4710-057, Braga, Portugal
5 CMEMS-UMinho, 4800-058, Guimarães, Portugal
6 Cellvax, SAS, 94800 Villejuif, France
7 Multidisciplinary Sepsis Group, Health Research Institute of the Balearic Islands (IdISBa), 07120 Palma de Mallorca, Spain
8 Centro de Investigación Biomédica en Red de Enfermedades Infecciosas (CIBER-INFEC), 28029 Madrid, Spain
9 Grup de Biotecnologia Molecular i Industrial, Departament d'Enginyeria Química, Universitat Politècnica de Catalunya, 08222 Terrassa, Spain
10 Basque Centre for Materials and Applications (BCMaterials), UPV/EHU, 48940 Leioa, Spain
11 IKERBASQUE, 48009, Bilbao, Spain
*Email: cesar.fernandez@csic.es

MATERIALS AND METHODS

Reagents and solutions. All reagents used were of high purity, analytical grade or equivalent. 2-(N-morpholino)ethanesulfonate hydrate (MES, ≥ 99.5%), 1-ethyl-3-(3-dimethylaminopropyl) carbodiimide hydrochloride (EDC), bovine serum albumin (BSA, lyophilized powder, ≥ 96%), PBS tablets, Tween 20, sodium azide (≥ 99.0%), H_2O_2 solution (30%) and ferrocenemethanol (97%) were purchased from Sigma-Aldrich (Madrid, Spain). All solutions were prepared using deionized water. 200-nm-diameter carboxylated magnetic nanoparticles (MNPs) (fluidMAG-ARA) were purchased from Chemicell GmbH (Berlin, Germany).

A 0.1 M MES buffer pH 5.0 was prepared by dissolving MES and adjusting the pH with 1 M NaOH. A 10 mM phosphate buffered saline (PBS) solution (NaCl 0.138 M; KCl 0.0027 M) pH 7.4 was obtained by dissolving commercial tablets. 0.1% BSA was dissolved in PBS to prepare PBS-BSA blocking buffer. Also, PBS was modified with 0.05% Tween 20, to prepare PBST buffer. A 0.1 M acetate buffer at pH 4.5 was prepared using acetic acid (≥ 99.7%, Panreac, Barcelona, Spain) and adjusting the pH with 1 M NaOH. 10 mM H_2O_2 and a 0.1 M ferrocenemethanol (in ethanol 96%) stock solutions were daily prepared. Artificial sputum medium was prepared as described elsewhere.[1] This solution contained an average concentration of the main chemical species present in real sputum.

MPO enzyme biomarker (100 µg, native human myeloperoxidase protein, ab91116) and MPO capture antibody (100 µg in PBS solution pH 7.4 containing 0.09% sodium azide, 1 mg/mL, Anti-MPO antibody [2C7], ab25989) were obtained from Abcam pl (Cambridge, UK). The MPO enzyme was reconstituted in 1 mL PBS solution and 100-µL aliquots were then prepared and stored at -20 ºC. The anti-MPO antibody was aliquoted (25-µL) and stored at -20 ºC. TNF-α and IL-8 biomarker assay reagents were included in the Human TNF-α ELISA (SEKA10602) and Human IL-8 ELISA (SEK10098) kits, purchased from Sino Biological Inc. (Beijing, China). These were the respective capture antibody solutions (in PBS solution pH 7.4), standard target analyte (lyophilized) and the detection antibody conjugated to HRP solutions (in PBS solution pH 7.4 containing 50% HRP-protector).

Electrochemical transducer. An array comprising five electrochemical cells of gold thin-film electrodes in a two-electrode cell arrangement was fabricated by a standard photolithographic/lift-off process on 4-inch silicon wafers at the IMB-CNM Clean Room facilities.[2] 8×25 mm^2 silicon chips, each one including five 1×1 mm^2 working electrodes (WE) and a single 13×1.5 mm^2 counter / reference electrode (CRE) shared by all the cells in the array, were manufactured. Thus, six contact pads were just included (Figure 1 A). The CRE exposed area was divided into five 1×1.5 mm^2 areas defined by the SiO_2 passivation layer openings. A detailed description of the array fabrication process and its analytical characterization has previously been published.[3] The array particular design minimizes the overall area of the silicon chip without compromising its electrochemical performance. It was demonstrated that it is highly suited for the detection of enzymes coupled to reversible redox mediators, whose activity produced a redox molecule in a solution containing the corresponding redox counterpart.

Before use, the chip was cleaned by immersion in a cold acid piranha solution for 2 min. Then, an electrochemical activation was carried out in a 0.5 M KNO_3 solution, where a cyclic potential scan from +0.2 to -2.2 V was individually applied to the five on-chip WEs (all of them short-circuited) and the CRE and repeated 25 times using an external platinum wire as CE (MicruX Technologies, Gijón, Spain) and the on-chip gold electrodes not being activated as pseudo-reference electrodes.

Fabrication of the paper fluidic component. The paper fluidic component was designed using computer-aided design software. It includes five 2-mm wide and 20-mm long fluidic channels, separated 1 mm, on a 15×22 mm^2 substrate. Channel patterning was carried out by wax printing using a Xerox ColorQube 8870 printer on a Whatman grade 1 chromatographic paper (GB HealthCare). The printed samples were placed on a hot plate at 100 ºC for 10 min after printing to allow the wax to penetrate the substrate all the way through to the opposite surface. This experimental step ensured the fabrication of barriers capable of isolating the liquids flowing on one channel to those flowing on adjacent ones. The same paper

material was used to define a 15×50 mm^2 wicking pad, which was folded and placed overlapping by 2.5 mm the front and back faces of the fluidic channels. Both paper pieces were arranged and kept in place by sandwiching them between two polyvinyl layers (150 µm-thick sign vinyl film from Metamark Ld., Lancaster, UK). The vinyl layers were patterned using a cutter plotter (Roland CX24 Camm-1 Servo Desktop Vinyl Cutter Plotter, Arista Sistemas Gráficos, Barcelona, Spain), to open several windows for exposing specific areas of the paper and also for facilitating the further alignment of the overall paper component with the electrochemical transducer array. A first window was defined on the vinyl front layer in order to open the sample addition areas of the five fluidic channels. A second window left the sink area uncovered for favoring the evaporation of the liquids arriving to the sink pad once they flow through the detection area of the device. A third window was opened at the vinyl back layer, to expose the detection areas of the paper channels so that they were in contact with the electrochemical cells. On both vinyl layers, additional windows were opened to get access to the electrical connectors. Four 1.5 mm diameter hollow circles were also defined at the corners of the paper component to facilitate the alignment with the transducer array. Figure S1 shows a detailed scheme of the parts that form the paper component.

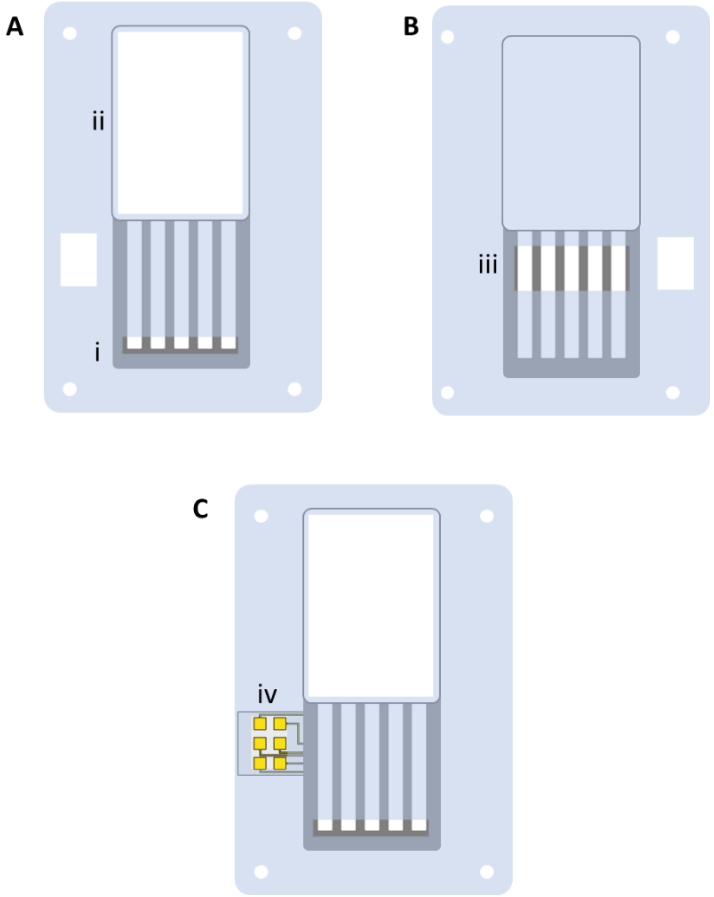

Figure S1. Layout of the paper fluidic component. A- Front Side; B- Back side; C- Alignment with the array chip. Opened windows are machined in the two vinyl layers to access the paper in the sample addition (i) and evaporation area (ii) in the front side, and the sensing area (iii) in the back side, as well as to allow contacting the on-chip contact pads (iv).

Assembly of electrochemical transducer and paper components. The electrochemical transducer array and the paper fluidic component were assembled in a PMMA cartridge fabricated in-house, in an arrangement depicted in Figure 1. The cartridge was designed with Corel Draw v.17 software and machined using a CO_2–laser cutter-engraver (Epilog Mini 24, Epilog Laser, Golden, CO, US) on 5-mm thick PMMA substrates. The bottom part of the cartridge defined a rectangular area where the transducer array was placed. Also, it included five 2-mm diameter neodymium magnets (Supermagnete, Gottmadingen, Germany), placed at a 1.5-mm distance from the WEs so that they were aligned with the five electrochemical

cells of the array. Moreover, four 1.5-mm diameter poles were incorporated to easily align the paper component over the transducer array. The cartridge lid was placed on top to firmly press the transducer against the paper component, keeping a constant pressure between them by means of four clamping structures. Two windows were opened on the lid to leave the sample addition and sink areas of the paper component uncovered. Also, a third window was defined where a 6-pin spring-loaded connector (Preci-dip SA, Delémont, Switzerland) was inserted to contact the pads of the transducer array. The lid also incorporated a 150-μm thick polyvinyl pad that was aligned over the detection areas of the device to push down the paper channels and thus ensure the intimate contact between the paper and the transducer. The cartridge was designed so that it could easily be assembled and disassembled to get access to both the transducer array and the paper component whether necessary as well as to facilitate the replacement of the paper component after one measurement. The dimensions of the thus-fabricated overall device were $48\times67\times19$ mm^3. Pictures of the components, the constructed device and scheme of its parts and how they are assembled are shown in Figure 1. Five individually-addressable paper-microfluidic electrochemical cells that allowed performing five quasi-simultaneous electrochemical measurements for multiplexed analytical purposes were thus defined.

Functionalization of MNPs. The MNPs were modified with the capture antibodies to the selected target biomarkers by covalent linkage between the MNP carboxylic groups and the antibody primary amines using the well-known carbodiimide chemistry.[4] The procedure was as follows. Firstly, 5 mg of MNPs were washed 2×1 mL MES buffer by suspension and concentration using a magnetic separator (MagnetoPURE, Chemicell GmbH). Following the second wash, the MNPs were resuspended in 125 μL MES buffer. Then, 5 mg of EDC were dissolved in 125 μL MES buffer and added to the MNPs. The suspension was mixed gently at 750 rpm for 15 min at room temperature (RT) using a circular shaker (MS 3 digital, IKA GmbH, Staufen, Germany). Then, the MNPs were washed with 2×1 mL MES buffer and resuspended immediately in 125 μL MES buffer containing 25 μg of the capture antibody. After an incubation under stirring at 750 rpm for 2 h at RT, the

modified MNPs were washed 3×1 mL PBS. The supernatants of the antibody incubation and washing steps were collected and analyzed by Bradford method.[5] Next, a blocking step was performed by incubating the functionalized MNPs in 200 µL PBS-BSA blocking buffer under stirring at 750 rpm for 2 h at RT. Finally, the functionalized MNPs were concentrated and then resuspended in 200 µL of 10 mM PBS containing 0.05% sodium azide and stored in the refrigerator at 4 ºC, until use.

Magneto-immunoassays for the three selected biomarkers. The optimized conditions for the magneto-immunoassays were achieved by performing 2D assays in round-bottom 96 microplates without any surface treatment. A series of functionalized MNPs dilutions were prepared in PBS and a constant concentration of the respective biomarker (750 pg/mL for TNF-α and 1000 pg/mL for IL-8) was added and incubated at 750 rpm for 30 min at RT. After that, the MNPs were washed three times with 200 µL PBST solution, by placing the microplate on a magnetic rack, allowing the MNPs to migrate to the magnet until the liquid was clear (approximately 1 min) and then removing the supernatant. Three different concentrations of each specific detection antibody labelled with HRP (0.5, 1 and 2 µg/mL) in PBST were tested to determine the best conditions for the magneto-immunoassays. The Ab-HRP was incubated at 750 rpm for 30 min at RT. After an additional washing step, a 100 µL/well substrate solution was added and incubated at 750 rpm for 30 min at RT. After collecting all the supernatants, color development was stopped with 50 µL/well of 4 N H_2SO_4 solution, and the absorbance was read at 450 nm with a SpectramaxPlus microplate spectrophotometer controlled by SoftmaxPro v4.7 software from Molecular Devices (Sunnyvale, CA, USA). In the case of MPO, the 2D assay was not performed as the Ab-HRP was not required and the concentration of MNPs for the MPO magneto-immunoassay was the same to the one previously optimized for the other two biomarkers.

Once the optimal concentrations of MNPs and Ab-HRP for each biomarker were selected, the measurements for estimating the analytical performance were carried out in two consecutive days by using Eppendorf tubes. The selected concentrations of functionalized MNPs were prepared in each tube, and then incubated with the

biomarker standard solutions (from 0 to 3000 pg/mL in PBST for TNF-α; from 0 to 2000 pg/mL in PBST for IL-8; from 10 to 2000 ng/mL in PBST for MPO, 50 µL/tube). After 30 min of incubation at RT under gentle stirring (750 rpm), nanoparticles were washed with PBST (400 µL, 3 times) using a 12-tube magnetic separator rack (MagnaRackTM Cat. No. CS15000, Invitrogen, Carlsbad, CA, US) and resuspended in a solution of the specific detection antibody conjugate (2 µg/mL in PBST for each Ab-HRP, 100 µL/tube) in the case of TNF-α and IL-8. After 30 min of incubation under the same conditions described before, the MNPs were washed again and a substrate solution was added. As described for the 2D assays, after collecting all the supernatants, color development was stopped with 50 µL of a 4 N H_2SO_4 solution and the absorbance was read at 450 nm.

Analytical performance of the electrochemical device. All studies were performed at RT. An EmStat potentiostat with integrated MUX8-R2 multiplexer (Palmsens BV, Houten, The Netherlands) controlled by PSTrace v5.5 software, was used for all voltammetric and amperometric measurements. The electrochemical measurements were based on the detection of the electrodic reactions undergone by the ferrocenemethanol/ferrocinium-methanol (Fc-MeOH/$[Fc\text{-}MeOH]^+$) redox pair. Ferrocenemethanol was used as the redox mediator to measure the activities of both the HRP label and MPO biomarker. First, cyclic voltammetric experiments were performed with the whole device in a acetate buffer solution containing both Fc-MeOH and $[Fc\text{-}MeOH]^+$ cation in order to characterize the reversible redox process. This solution was prepared as follow: a 0.1 M acetate buffer pH 4.5 solution containing 2 mM Fc-MeOH, 0.3 mM H_2O_2 and 300 ng/mL MPO was incubated at room temperature under stirring at 750 rpm for 15 min. Using its peroxidase activity, the MPO enzyme catalyzes the reduction of H_2O_2 using the Fc-MeOH as electron donor, in-situ generating its redox counterpart $[Fc\text{-}MeOH]^+$. Considering the enzyme activity (180-220 U/mg protein) and the stoichiometry of the reaction, the MPO would consume all the H_2O_2 in solution. Therefore, the solution would roughly contain 1.4 mM Fc-MeOH and 0.6 mM $[Fc\text{-}MeOH]^+$ cation. Following the incubation step, 5 µL of the solution were added to the sample pad of one paper

channel. After 1 min flowing, the solution reached the electrochemical cell and the cyclic voltammogram was performed at 50 mV/s.

The analytical measurements of the three biomarkers comprised the following steps:

1. Biomarker capturing. The immunoassay reactions required for the selective detection of the different biomarkers were carried out in 1.5-mL Eppendorf tubes using the concentrations set up in the optimization of the corresponding magneto-immunoassays (previous section). However, the number of steps and assay time were reduced by carrying out the simultaneous incubation of the sample with the functionalized MNPs and the HRP-labelled detection antibody for 1 h. Five assays could be carried out at once, considering the five channels of the device. It should be noted that one sample aliquot was required for each channel. That is, each one was incubated in an individual tube containing MNPs modified with the capture and detection antibody (when necessary) for just one biomarker. Once the immunoreactions took place, the MNPs were trapped with the aid of an external magnet, washed three times with 200 µL of PBST and resuspended in 100 µL PBST (Figure S2 B) before being added to one channel of the device.
2. MNP flowing and trapping on the device. Sequential additions of the MNP suspensions prepared in individual tubes were carried out on the different paper channels of the device. For each individual paper channel, 5 µL of one MNP suspension was added to the sample addition area and left it to flow by capillary action till reaching an area located 1.5 mm upstream the device detection area where the MNPs were trapped by the integrated magnet located underneath the paper channel. Then, 5 µL of PBST was added to ensure that all the added MNPs effectively flowed and were trapped by the magnet, followed by the addition of 5 µL of acetate buffer solution that washed away any unbound MPO or HRP-antibody conjugate that might have remained in the Eppendorf tubes after the washing steps. Each solution was left to flow completely on the paper channel by capillary action before the addition of the following one and this took around 4 min (Figure S2 C).

3. Enzyme reaction and electrochemical measurement. Finally, 5 μL of a freshly prepared solution containing 2 mM Fc-MeOH and 0.3 mM H_2O_2 in acetate buffer were added to each paper channel and allowed to flow for 3 min. Then, chronoamperometric measurements of the five electrochemical cells were carried out consecutively. The potential set for these measurements was chosen from the cyclic voltammetric experiments mentioned above. The integrated multiplexer of the potentiostat included 5 channels, each one connected to one cell of the transducer array. This enabled the polarization of a WE while leaving the rest of them at open circuit. Each measurement was carried out for 3 s, recording the current intensity every 200 ms. Thus, the overall analysis time was 15 s for the five electrochemical cells. The current responses at 1.6 s were used as the analytical signal (Figure S2 C).

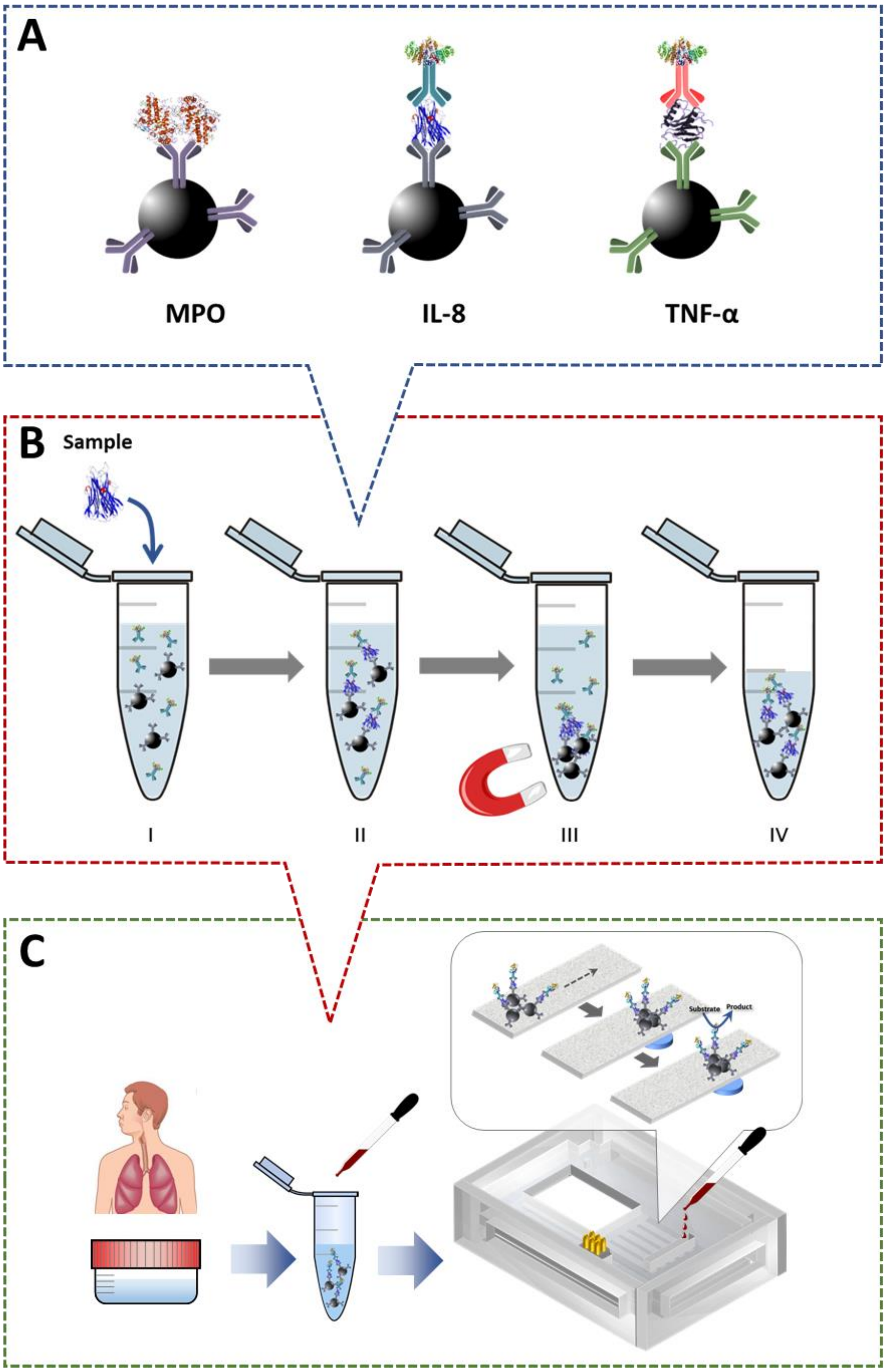

Figure S2. Magneto-immunoassays and device electrochemical readout. (A) Scheme of the immunoassays for the three target biomarkers. Detection of MPO was based on a direct format using an anti-MPO capture antibody immobilized on the MNPs. Detection of IL-8 and TNF-α was based on a sandwich-like format using detection antibodies conjugated to HRP enzyme. (B) Sample pretreatment for biomarker capturing: (I) Sample is incubated with the functionalized MNPs and the HRP-labelled detection antibody, (II) The immunoreactions take place, (III) The MNPs are trapped with an external magnet and

washed with PBST and (IV) the MNPs are resuspended in 100 µL PBST. (C) Overall device analytical performance: sample collection and pretreatment followed by addition of some 5 µL of the MNPs suspension to the sample addition area of each paper channel. Inset: MNPs flow by capillary action till they are trapped by the integrated magnet. After the washing step of the paper channel, 5 µL of a freshly prepared solution containing 2 mM ferrocenemethanol and 0.3 mM H_2O_2 in acetate buffer are added and allowed to flow for 3 min. Then, chronoamperometric measurements of the five electrochemical cells are carried out. Schemes in Figures S2 B and C, show the assay steps for IL8. Objects are not to scale.

HRP label and MPO enzymes catalyzed the reduction of H_2O_2 using the Fc-MeOH as electron donor, in-situ generating the $[Fc\text{-}MeOH]^+$ redox counterpart. Using 0.3 mM H_2O_2 and considering the stoichiometry of the reaction, the enzymes would consume 0.6 mM Fc-MeOH should H_2O_2 be completely consumed. Therefore, by adding 2 mM Fc-MeOH, an excess of this redox species in solution would always be guaranteed, which is required for the correct performance of the two-electrode electrochemical cell arrangement defined on the array. We refer to our previous publication for more details about this.[3] Under these experimental conditions, the $[Fc\text{-}MeOH]^+$ generated during the enzymatic process could be reduced back to Fc-MeOH. The corresponding catalytic and electrode redox reactions are shown in Figure 2 A. The faradaic current recorded was stoichiometrically related to the amount of enzyme, and consequently, to the concentration of biomarker (IL-8, TNF-α or MPO) in solution. Calibration curves were produced in triplicate for each biomarker by using PBST and artificial sputum as sample matrixes.

Human sputum samples. Eight real sputum samples were collected by the Department of Microbiology from Son Espases University Hospital (Palma, Balearic Islands). All samples were anonymized leftover specimens that, otherwise, would have been discarded. Any leftovers were destroyed. For these reasons, the institutional review board considered the study as minimal-risk research and waived the requirement for informed consent (Ethics and Scientific Committee approval with reference IB 4005/19-PI).

Of these samples, three were induced sputum specimens from healthy controls. The remaining five samples were sputum specimens from patients diagnosed with COPD and with exacerbated symptoms. All samples were kept at -20 ºC until used. After thawing at RT, they were pre-treated in order to extract the biomarkers. For the analysis of IL-8 and TNF-α, weighted sputum specimens were placed in a 1.5-mL Eppendorf tube (Table S1), vortexed vigorously and incubated with a 2×volume (according to the sputum weight) of 0.2% dithiothreitol (DTT, Sigma Aldrich) in 10 mM PBS solution at 750 rpm for 30 min to homogenize the sample. DTT is a reducing mucolytic agent that cleaves disulphide bonds of mucoproteins, disrupting the mucus and decreasing sputum viscosity.[6] Subsequently, with the aim of minimizing the matrix effect, the treated sample was diluted 1:2 in 10 mM PBS and centrifuged at 6000 rpm for 20 min to get rid of possible particles that had not been resuspended.

However, as a strong reducing agent, DTT may disrupt the stable structure of the MPO enzyme, resulting in a decrease of its catalytic activity,[7] which would seriously affect the enzyme electrochemical detection. Therefore, for the analysis of MPO, the fluid-phase of the sputum was used.[8] For that, weighted sputum specimens were placed in a 1.5-mL Eppendorf tube (Table S1) and incubated with a 8×volume PBS in an ice bath at 750 rpm for 15 min, rocking for 15 s every 5 min. The mixture was centrifuged at 2000 rpm for 10 min. Then, the supernatant was collected and filtered through a 0.22 μm filter to remove any bacteria and particulate material that may be present.

The resulting sample extracts were then used for the analysis of the three biomarkers with the electrochemical device.

Table S1. Details of the analyzed sputum samples. Weighted sputum amounts of the different specimens for the extraction of the biomarkers.

Sample	Extracted with PBS for MPO (mg)	Treated with DDT for IL-8 and TNF-α (mg)
**HI 1**	105	166
**HI 2**	112	150
**HI 3**	110	174
**COPD 1**	117	142
**COPD 2**	112	130
**COPD 3**	106	158
**COPD 4**	173	134
**COPD 5**	164	134

Statistical analysis. The semilogarithmic curves commonly recorded when working with immunoassays and obtained for the three biomarkers in both the optimization of the magneto-immunoassay experiments and the electrochemical device were generated by fitting data points using the GraphPad Prism v5.03 software (GraphPad Software Inc., San Diego, CA, USA), which also provides the R^2 and EC_{50} values. The LD is calculated using the 3σ IUPAC criterion. Results were represented as mean values (standard deviation) of a number of replicates per experiment (N), as indicated in the figure and table legends. The analytical signals obtained for the human sputum samples were compared via an unpaired *t* test, considering $P < 0.05$ to be statistically significant.

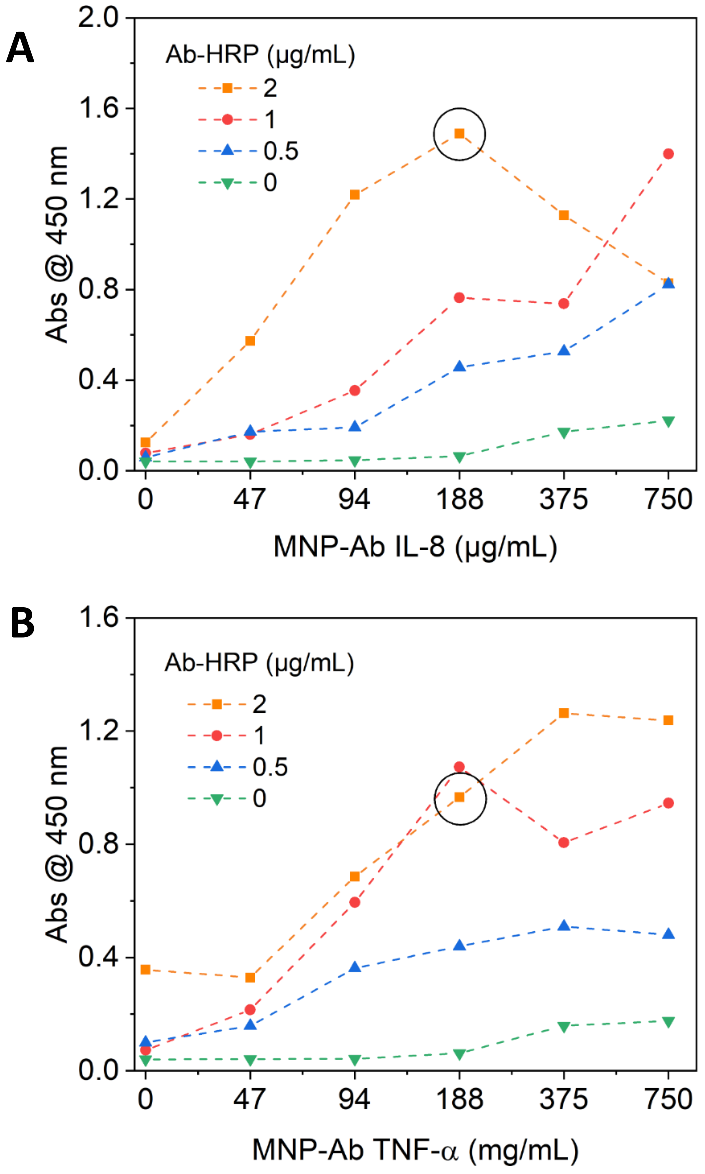

Figure S3. Optimization of the magneto-immunoassays with optical detection. Concentration of MNPs and Ab conjugates for (A) IL-8 and (B) TNF-α assays. TMB was used as redox mediator and the absorbance was measured at 450 nm.

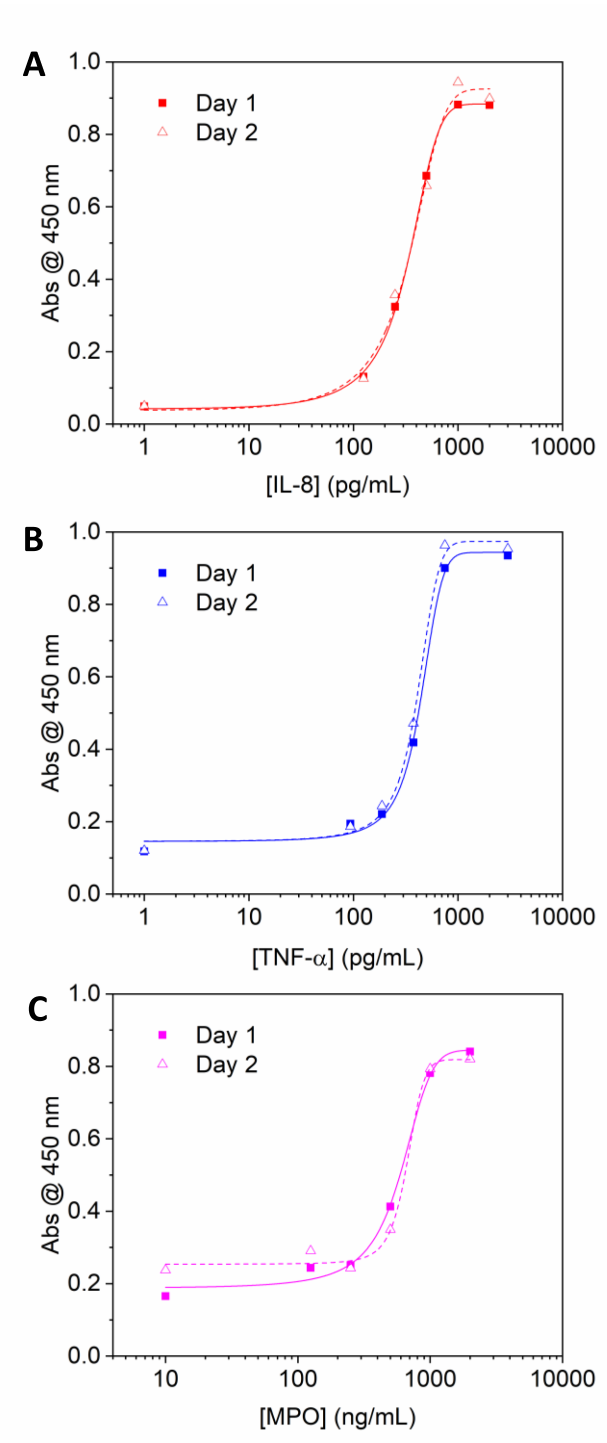

Figure S4. Dose-response curves of the magneto-immunoassays performed in two consecutive days. Plots obtained under the optimized experimental conditions (Figure S3) for (A) IL-8 and, (B) TNF-α. (C) Calibration curve for MPO using the same experimental conditions as for IL-8 and TNF-α. TMB was used as redox mediator and the absorbance was measured at 450 nm. The dose-response semi-logarithmic curves were fitted using the GraphPad Prism software.

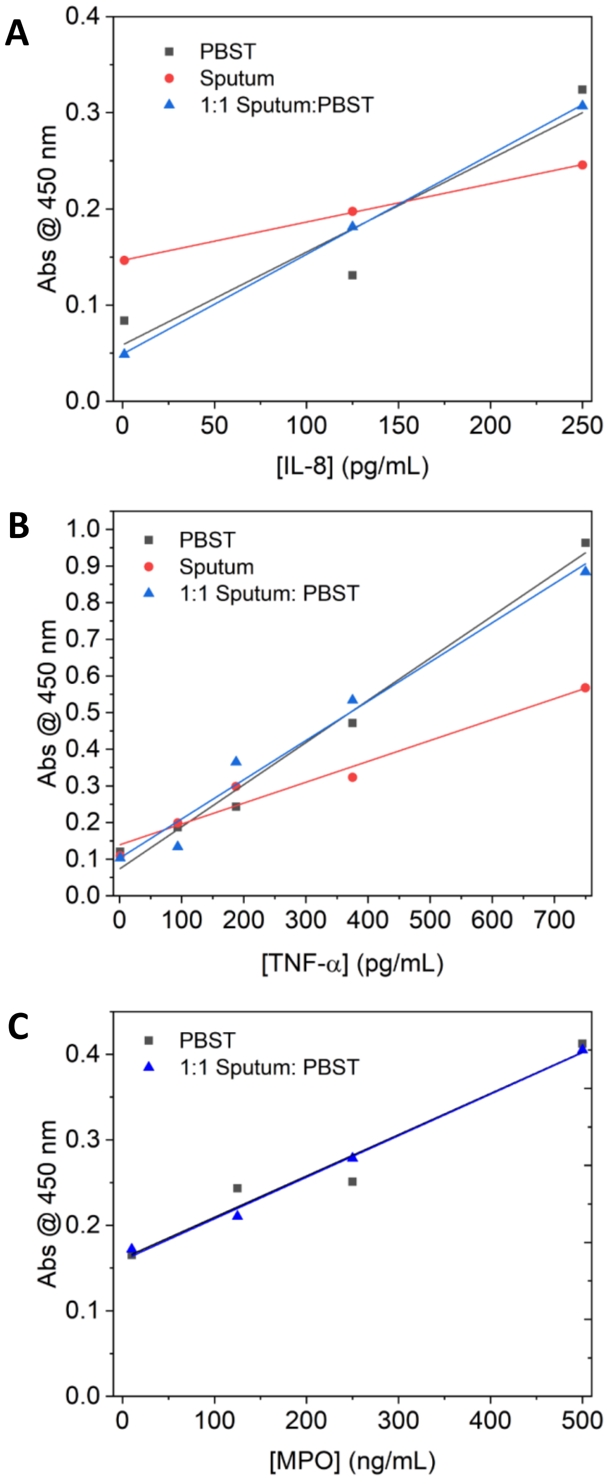

Figure S5. Study of matrix effects. Absorbance measurements carried out with solutions containing different concentrations of (A) IL-8 and, (B) TNF-α prepared in the PBST working buffer, artificial sputum and 1:1 dilution of artificial sputum in PBST. (C) Similar study performed for MPO but with just the 1:1 dilution of the artificial sputum after observing the effect of this dilution on the detection of IL-8 and TNF-α. TMB was used as redox mediator and the absorbance was measured at 450 nm.

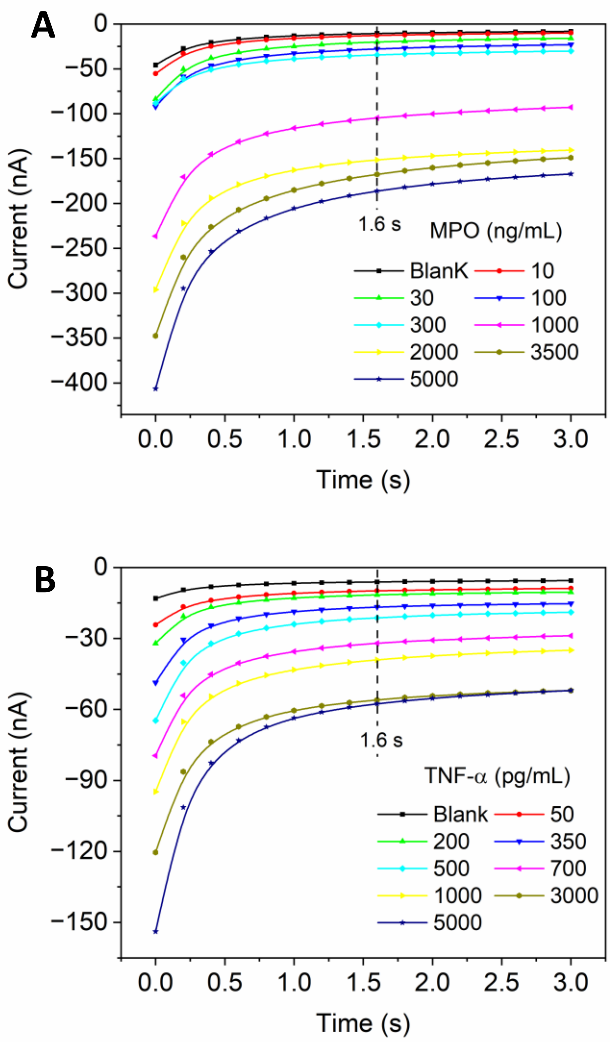

Figure S6. Raw chronoamperometric responses recorded with the electrochemical device for the detection of (A) MPO and (B) TNF-α. The concentration ranges were from 0 to 5000 ng/mL for MPO, and from 0 to 5000 pg/mL for TNF-α. The current responses recorded at 1.6 s were used as analytical signal.

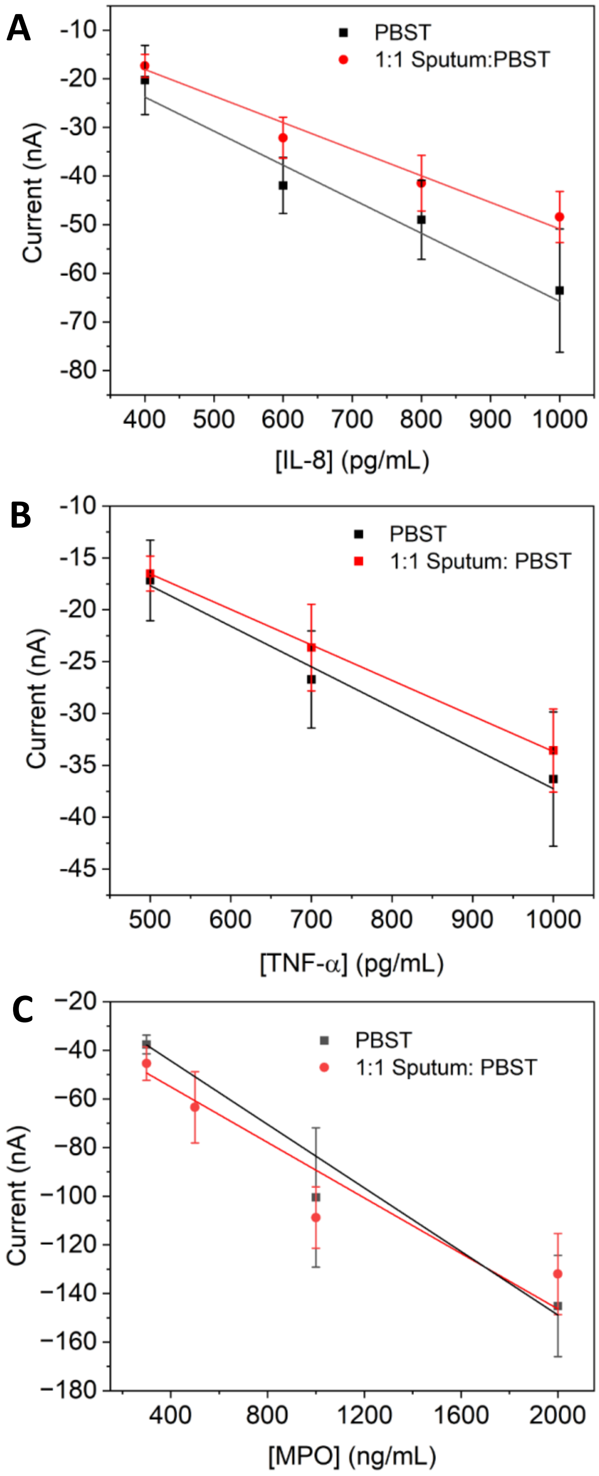

Figure S7. Study of the matrix effect on the electrochemical device. Electrochemical measurements carried out with solutions containing different concentrations of (A) IL-8, (B) TNF-α and, (C) MPO, prepared in the PBST working buffer and in a 1:1 dilution of the artificial sputum solution in PBST.

Table S2. Estimated concentration values of each biomarker in the human sputum samples. The concentration values were estimated by interpolation using the recorded analytical signals from Figure 4 and the respective calibration curves, also considering the weight of sputum sample used to obtain each extract (Table S1). The standard deviation values of three replicates are indicated in brackets.

Sample	[MPO] (ng/mg sputum)	[IL-8] (pg/mg sputum)	[TNF-α] (pg/mg sputum)
COPD 1	5 (2)	5.0 (0.6)	7 (2)
COPD 2	4.4 (0.7)	4.7 (0.7)	5 (1)
COPD 3	6.1 (0.9)	3.6 (0.4)	4.8 (0.4)
COPD 4	3.1 (0.5)	3.9 (0.2)	4.8 (0.6)
COPD 5	2.4 (0.2)	4.7 (0.4)	3.8 (0.8)

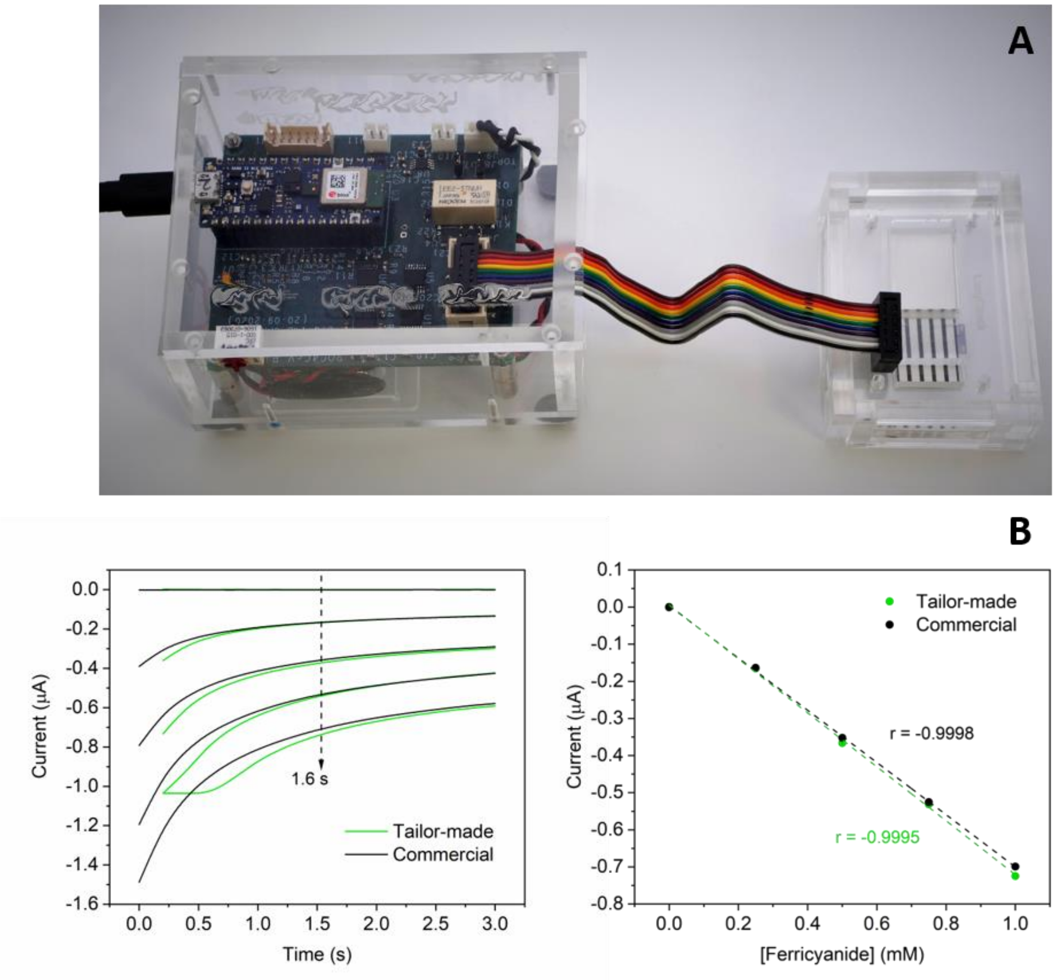

Figure S8. (A) Photograph of the electrochemical device connected to the battery-powered electronics made in house for carrying out the simultaneous chronoamperometric measurement of the five on-chip electrochemical cells. (B) Comparative chronoamperometric study carried out with the tailor-made electronics and the commercial one in 0.1 M solutions containing 2 mM ferrocyanide and different concentrations of ferricyanide (0, 0.25, 0.5, 0.75 and 1 mM). The recorded chronoamperometric responses are shown together with the calibration curves plotted using the current recorded at 1.6 s, as done throughout this work. A very small bias is observed specially during the first second of the signal recording that is related to how the tailor-made instrumentation works in contrast with the commercial one. However, this bias becomes very small at longer times as it happens to be at the current used as the analytical signal of 1.6 s. This has a negligible effect on the sensitivity of the electrochemical device, as observed in the calibration curves.

References

(1) Kirchner, S.; Fothergill, J. L.; Wright, E. A.; James, C. E.; Mowat, E.; Winstanley, C. Use of Artificial Sputum Medium to Test Antibiotic Efficacy against Pseudomonas Aeruginosa in Conditions More Relevant to the Cystic Fibrosis Lung. *J. Vis. Exp.* **2012**, *64*, No. e3857.

(2) Orozco, J.; Suárez, G.; Fernández-Sánchez, C.; McNeil, C.; Jiménez-Jorquera, C. Characterization of Ultramicroelectrode Arrays Combining Electrochemical Techniques and Optical Microscopy Imaging. *Electrochim. Acta* **2007**, *53*, 729–736.

(3) Gutiérrez-Capitán, M.; Baldi, A.; Merlos, Á.; Fernández-Sánchez, C. Array of Individually Addressable Two-Electrode Electrochemical Cells Sharing a Single Counter/Reference Elec-trode for Multiplexed Enzyme Activity Measurements. *Biosens. Bioelectron.* **2022**, *201*, No. 113952.

(4) Chemicell GmbH. Protocol A10. http://www.chemicell.com/products/protocols/docs/fluidMAG-ARA.pdf (accessed Jun 13, 2022).

(5) Bio-Rad. Bio-Rad Protein Assay. https://www.bio-rad.com/webroot/web/pdf/lsr/literature/LIT33.pdf (accessed Jun 13, 2022).

(6) Woolhouse, I. S.; Bayley, D. L.; Stockley, R. A. Effect of Sputum Processing with Dithiothreitol on the Detection of Inflammatory Mediators in Chronic Bronchitis and Bronchiectasis. *Thorax* **2002**, *57*, 667–671.

(7) Grebski, E.; Peterson, C.; Medici, T. C. Effect of Physical and Chemical Methods of Homogenization on Inflammatory Mediators in Sputum of Asthma Patients. *Chest* **2001**, *119*, 1521–1525.

(8) Wang, F.; Liang, Z.; Yang, Y.; Zhou, L.; Guan, L.; Wu, W.; Jiang, M.; Shi, W.; Deng, K.; Chen, J.; Chen, R. Reproducibility of Fluid-Phase Measurements in Pbs-Treated Sputum Supernatant of Healthy and Stable COPD Subjects. *Int. J. COPD* **2019**, *14*, 835–852.
